# The multi-faced role of FUNDC1 in mitochondrial events and human diseases

**DOI:** 10.3389/fcell.2022.918943

**Published:** 2022-07-25

**Authors:** Nannan Tan, Tianhua Liu, Xiaoping Wang, Mingyan Shao, Miao Zhang, Weili Li, Guanjing Ling, Jinchi Jiang, Qiyan Wang, Jing Li, Chun Li, Wei Wang, Yong Wang

**Affiliations:** ^1^ School of Chinese Medicine, Beijing University of Chinese Medicine, Beijing, China; ^2^ Modern Research Center for Traditional Chinese Medicine, Beijing University of Chinese Medicine, Beijing, China; ^3^ School of Life Sciences, Beijing University of Chinese Medicine, Beijing, China; ^4^ School of Chinese Materia Medica, Beijing University of Chinese Medicine, Beijing, China; ^5^ School of Basic Medicine, Guangzhou University of Chinese Medicine, Guangzhou, China

**Keywords:** FUNDC1, mitophagy, mitochondrial fission, endoplasmic reticulum, I/R injury

## Abstract

Mitophagy plays a vital role in the selective elimination of dysfunctional and unwanted mitochondria. As a receptor of mitophagy, FUN14 domain containing 1 (FUNDC1) is attracting considerably critical attention. FUNDC1 is involved in the mitochondria fission, the clearance of unfolded protein, iron metabolism in mitochondria, and the crosstalk between mitochondria and endoplasmic reticulum besides mitophagy. Studies have demonstrated that FUNDC1 is associated with the progression of ischemic disease, cancer, and metabolic disease. In this review, we systematically examine the recent advancements in FUNDC1 and the implications of this protein in health and disease.

## 1 Introduction

Mitochondria regarded as the direct descendants of a bacterial endosymbiont are symbiotic with the host cell (Gray et al., 1999). To the best of our knowledge, approximately 99% of proteins used for maintaining mitochondrial function are regulated by nuclear genes, while only 13 proteins are coded by mitochondria (Song et al., 2021). Apart from providing energy for the host cell, mitochondria can sequester hazardous substances by creating an isolated space in cells (Song and Dorn, 2015). Furthermore, mitochondria are vulnerable when they participant in generating reactive oxygen species (ROS), iron metabolism, and lipid oxidation. Accumulation of damaged and superfluous mitochondria are detrimental to cells and organs (Liu et al., 2014; Zorov et al., 2014), suggesting that the process of clearance of mitochondria should not be overlooked.

Mitophagy that responsible for the selective elimination of dysfunctional and unwanted mitochondria was observed for the first time under electron microscopy by glucagon-stimulated stem cell activation (Deter and De Duve, 1967; Hirota et al., 2015). The past few years have witnessed an explosion in our understanding of mitophagy, which preserves mitochondrial function and cell homeostasis under diverse (patho-)physiological conditions such as hypoxia, starvation, and exposed cold stimulus (Hirota et al., 2015; Liu et al., 2021a).

The molecular mechanisms of mitophagy include PTEN induced putative kinase 1 (PINK1)-Parkin pathway and receptor-mediated pathway. PINK1-Parkin partnership mainly modulates the turnover of depolarized mitochondria (Springer and Macleod, 2016). When the mitochondrial membrane potential decreases, PINK1 firstly accumulates in the mitochondrial outer membrane, and then recruits Parkin, which is an E3 ubiquitin ligase, to the mitochondrial outer membrane, leading to the ubiquitination of various proteins in the mitochondrial outer membrane (Yoshii et al., 2011). Simultaneously, the autophagy receptor optineurin, which binds to light chain 3 (LC3), is recruited to ubiquitinated mitochondria and located to the outer membrane surface by phosphorylation of TANK-binding kinase 1. And then, the damaged mitochondria are engulfed into autophagy precursors, which are ultimately degraded by the conserved lysosomal pathway (Evans and Holzbaur, 2020).

Mitophagy receptors include BCL2 interacting protein three like (BNIP3L/NIX) and FUNDC1. FUNDC1mainly participates in the regulation of mitochondrial homeostasis under hypoxic (Liu et al., 2012a). Missed or mutated FUNDC1 may promote the progression of human disease. For example, the loss of FUNDC1 leads to mitochondrial fragmentation, and fails to repair infarcted hearts during the differentiation of cardiac progenitor cells (Lampert et al., 2019). Geng Guangfeng and his colleagues found that intervening FUNDC1 could simultaneously improve renal anemia and renal fibrosis (Geng et al., 2021). Moreover, Lei Liu and his colleagues gave a detailed description of the role of FUNDC1-mediated mitophagy in cardiovascular diseases (Liu et al., 2021b). Instances of disease ranging from ischemic disease to cancer, as well as metabolic related disease, could also be influenced by FUNDC1. Thus, this review aims to delineate the timely advancement of FUNDC1 and its role in human disease.

## 2 The structure of FUNDC1

FUNDC1 is one of the paralogous subfamilies of FUN14 domain-containing protein family which is present in eukaryotes, archaea, and bacteria (Wu et al., 2017a). Human FUNDC1 is widely expressed in the body, especially in the heart (Zhang et al., 2017). It contains 155 amino acids and is mainly located in the outer membrane of mitochondria. Furthermore, FUNDC1 has three transmembrane regions, the N-terminal is exposed to the cytoplasm. The exposed part includes a LC3-interacting region (LIR, Y18-E-V-L21) which can bind to LC3 to regulate the occurrence of mitophagy (Liu et al., 2012a; Wu et al., 2017a). Eleven lysine residues sites such as K70 and K119, which are included in the transmembrane region of FUNDC1, can bind to optic atrophy 1 (OPA1) and MARCH5, respectively (Chen et al., 2017a; Wu et al., 2017a).

## 3 The regulatory proteins of FUNDC1-mediated mitophagy

The mRNA level of FUNDC1 is downregulated under hypoxia condition (Wu et al., 2017a), but the specific transcriptional regulators of FUNDC1 are not deeply understood. As a nuclear transcription factor, nuclear transcription factor 1(NRF1) can participate in the activation of mitochondrial genes and transcription and translation of mtRNA (Carraway et al., 2010). After being activated by the peroxisome proliferator-activated receptor gamma coactivator 1alpha (PGC1α) and acetaldehyde dehydrogenase 2 (ALDH2), NRF1 can directly bind to 5’ promoter of FUNDC1, upregulating the level of FUNDC1, and further promoting mitophagy and mitochondrial biogenesis to maintain the normal function of mitochondria and cells (Li et al., 2020; Liu et al., 2021a). Moreover, FOXO3a that is a member of the fork head box class O (FOXO) family that are widely expressed transcription factors can regulate a variety of cellular physiological processes by targeting effector genes (Liu et al., 2018) and activate FUNDC1 to promote the occurrence of mitophagy in ISO-induced myocardial hypertrophy model (Liu et al., 2021c).

In normal condition, the LIR region of FUNDC1 is phosphorylated at Y18 and S13 sites by Src and CK2 kinase, respectively, to maintain inactivation. However, under long-term hypoxia condition, FUNDC1 is dephosphorylated at Y18 and S13 due to the inactivation of Src and CK2 kinase, leading to the occurrence of mitophagy (Liu et al., 2012a; Chen et al., 2014; Zhou et al., 2018a). MARCH5, an E3 ubiquitin ligase, is an important regulator of FUNDC1-mediated mitophagy. Interaction between MARCH5 and FUNDC1 means a crosstalk between ubiquitin- and mitophagy. Chen et al. (2017a), Chen et al. (2017b) suggested that MARCH5 homo-oligomers were disassembled under the early stage of hypoxia, and then MARCH5 bound to K119 site of FUNDC1, thereby making FUNDC1 ubiquitinated and degraded, which inhibiting mitophagy and avoiding improper clearness of undamaged mitochondria. As a negative factor for FUNDC1, micro-137 can directly target to FUNDC1 and then inhibit mitophagy (Li et al., 2014).

**FIGURE 1:**
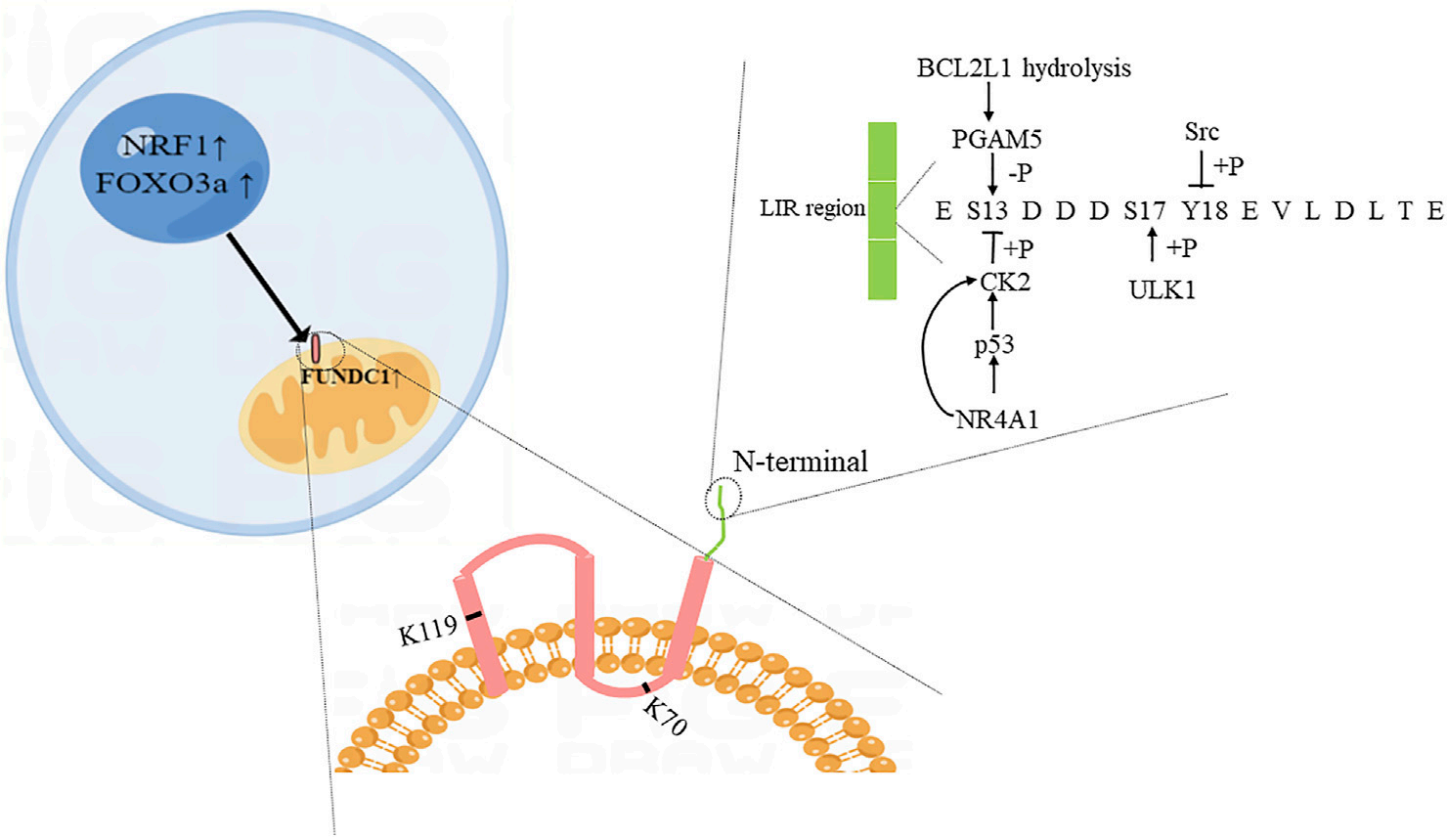
Diagram of possible structure and regulatory proteins of FUNDC1. The illustration was created by Figdraw (www.Figdraw.com).

Phosphoglycerate mutase family member 5 (PGAM5) exists in the inner membrane of mitochondria and protects PINK1 from degradation. After being stimulated by carbonyl cyanide *m*-chlorophenyl hydrazine in models, PINK1 can migrate from the inner membrane to the outer membrane under the protection of PGAM5 and bind with Parkin to complete the subsequent mitophagy process. The loss of PGAM5 affects the occurrence of PINK1-mediated mitophagy (Lu et al., 2014). Similarly, PGAM5 is important to regulate FUNDC1-mediated mitophagy. Under normal condition, BCL2-like 1 (BCL2L1/Bcl-xL) binds to PGAM5 to inhibit the activation of PGAM5. While under hypoxia condition, BCL2L1 is hydrolyzed, and PGAM5 is released. Activated PGAM5 dephosphorylates FUNDC1 at S13 and promotes the interaction between FUNDC1 and LC3, thereby inducing mitophagy (Wu et al., 2014a; Kuang et al., 2016; Biswal et al., 2018). In addition, FUNDC1 is phosphorylated by unc-51 like autophagy activating kinase 1 (ULK1) at S17 site under hypoxic condition (Wu et al., 2014b). Wang Li and his colleagues found that mitophagy that was activated *via* ULK1-FUNDC1 pathway could prevent nerve cells from apoptosis (Wang et al., 2018). Specific details of factors are shown in Figure 1.

## 4 FUNDC1-mediated mitochondrial events

### 4.1 Mitochondrial fission

Mitochondrial fission is strictly monitored in cells that can produce healthy mitochondria and induce mitophagy through the ROS produced by the division (Archer, 2013; Song and Dorn, 2015). It also participates in cytochrome C-mediated apoptosis pathologically (Cassidy-Stone et al., 2008). Dynamin-related protein 1 (DRP1, also known as DNM1L), which is recruited to the outer membrane of mitochondria, is essential to drive mitochondrial fission (Cerveny et al., 2007). In addition, as a dynamin-like guanosine triphosphatase, OPA1, which can be found as long-OPA1(L-OPA1) and short-OPA1(S-OPA1) forms, is located in the inner membrane of mitochondria and is vital for balancing mitochondrial fusion and fission (Wai et al., 2015). Seminal findings from Chen Ming and colleagues depicted a new molecular mechanism of mitochondria fission, which depended on the interaction between FUNDC1 and DNM1L, OPA1. Under normal condition, FUNDC1 interacted with OPA1 at the site K70. But OPA1 could be cleaved or degraded by yeast mitochondrial escape 1-like (YME1L) and OMA1 under mitochondrial stress condition. Then FUNDC1 and OPA1 separated, dephosphorylated FUNDC1 recruited DNM1L toward mitochondria, thus promoting mitochondrial fission (Chen et al., 2016).

Mitochondria-associated ER membranes (MAMs) are critical for the occurrence of mitochondrial fission. Under hypoxia condition, FUNDC1 is accumulated at MAMs by interacting with calnexin. As mitophagy proceeds, the association between FUNDC1 and calnexin becomes attenuated, and FUNDC1 recruits DRP1 toward mitochondria to complete mitochondrial fission (Wu et al., 2016a; Wu et al., 2016b). Mitochondrial fission and mitophagy are closely relevant. They seemed to be a continuous process, just as van der Bliek AM described in his paper (van der Bliek, 2016). Ubiquitin-specific peptidase 19 (USP19), identified as a deubiquitylase, is also an important regulator involved in MAMs-mediated mitochondrial fission (Volkmar et al., 2016). Under hypoxia condition, USP19 is recruited to ER, which is mainly responsible for removing the ubiquitin chain from FUNDC1. Then deubiquitinated FUNDC1 is stabilized at MAMs and recruits DRP1 toward the contact site to finish mitochondria fission (Chai et al., 2021). Under hypoxia condition, why does FUNDC1 interact with calnexin and then dissociate? Does this process recruit USP19 to MAMs or recruit DRP1 to mitochondria to interact with FUNDC1? Is USP19 able to interact with calnexin? How do MARCH5 and USP19 coordinate the ubiquitinated fate of FUNDC1? There are still no clear answers to these questions. Mitochondrial fission is vital for mitophagy. In order to get a better understanding of mitophagy, it is necessary to conduct in-depth research on the upstream (Chakrabarti and Higgs, 2021). Therefore, the molecular mechanism of FUNDC1-mediated mitochondrial fission and mitophagy is worthy of further study.

### 4.2 Iron homeostasis in mitochondria

Iron is an essential component to maintain cellular metabolism and is crucial for the normal function of mitochondria because it participates in the electron transport chain (Huang et al., 2011; Dietz et al., 2021). Moreover, mitochondria as machineries where iron-sulfur clusters (ISCs) are assembled and exported are necessary for modulating iron metabolism in cells (Paul and Lill, 2015). Relative drugs are being exploited for treating human disease. For example, mitochondrially targeted deferoxamine (mitoDFO) impairs mitochondrial respiration and biogenesis of [Fe-S] clusters/heme in cancer cells, thereby suppressing the proliferation and migration of cancer cells (Sandoval-Acuña et al., 2021).

As a fundamental process in the cell, iron metabolism is closely related to mitophagy. Chiang et al. (2021) suggested that in the frataxin knockout mouse, mitophagy was enhanced when iron was accumulated in mitochondria, resulting in the dysfunction of mitochondria. But in pathogenic yeast *Candida glabrata* cells, mitophagy is also enhanced under iron-depletion condition (Nagi et al., 2016). The result of Schiavi Alfonso and his colleagues’ study was similar that iron depletion could induce mitophagy and then extended *C. elegans* lifespan (Schiavi et al., 2015). The studies mentioned above suggest that either iron deficiency or iron overload has an impact on mitophagy. Wu Hao and his colleagues found that if the machinery of ISCs was disrupted, iron regulatory proteins 1 (IRP1) would suppress the translation of Bcl-xL, causing PGAM5 separate from Bcl-xL, finally initiating FUNDC1-mediated mitophagy (Wu et al., 2020). Pei Zhaohui and his colleagues discovered that under short-term high fat intake, the deficiency of FUNDC1 induced metabolic and cardiac dysfunction through ferroptosis regulated by specificity protein 1 (SP1) -acyl-CoA synthetase long-chain family member 4 (ACLS4) axis (Pei et al., 2021). In short, the relationship between FUNDC1 and iron metabolism is close in cells.

### 4.3 Clearance of unfolded protein

Damaged or excess proteins can promote proteotoxic effects if they are not removed in time (Song, 2019). FUNDC1-mediated mitophagy is involved in the clearance of damaged proteins collaborating with ubiquitination. Under stress condition, FUNDC1 interacts with HSC70 belonging to the HSP70 family, which is a member of the heat shock protein (HSP)family (Hartl and Hayer-Hartl, 2002; Liu et al., 2012b), transporting misfolded proteins to mitochondria matrix, and the proteins are degraded in the presence of LonP1, which is a AAA protease. The formation of mitochondrion-associated protein aggregates (MAPAs) is triggered when the misfolded proteins are over-accumulation in the mitochondrial matrix. Then FUNDC1-FIS1-mediated mitophagy is activated to promote the clearance of unfolded proteins. If massive unfolded proteins are not timely disposed, cells will finally become senescent. Li et al. (2019a), Li et al. (2019b) pointed out that FUNDC1-LonP1 axis also played an important role in maintaining mitochondrial reprogramming and cellular plasticity in cancer cells (Cappellini et al., 2020). Wang Yue’s study confirmed that the coordination between FUNDC1-mediated mitophagy and unfolded proteins could attenuate inflammation-mediated myocardial injury in septic cardiomyopathy (Wang et al., 2021a).

### 4.4 Interaction with other organelles

The mitochondria generally maintain a relationship with other organelles in the cells. Such interactions could offer some forms of benefit. FUNDC1 plays a key role in the crosstalk between mitochondria and other organelles.

#### 4.4.1 Endoplasmic reticulum

**FIGURE 2:**
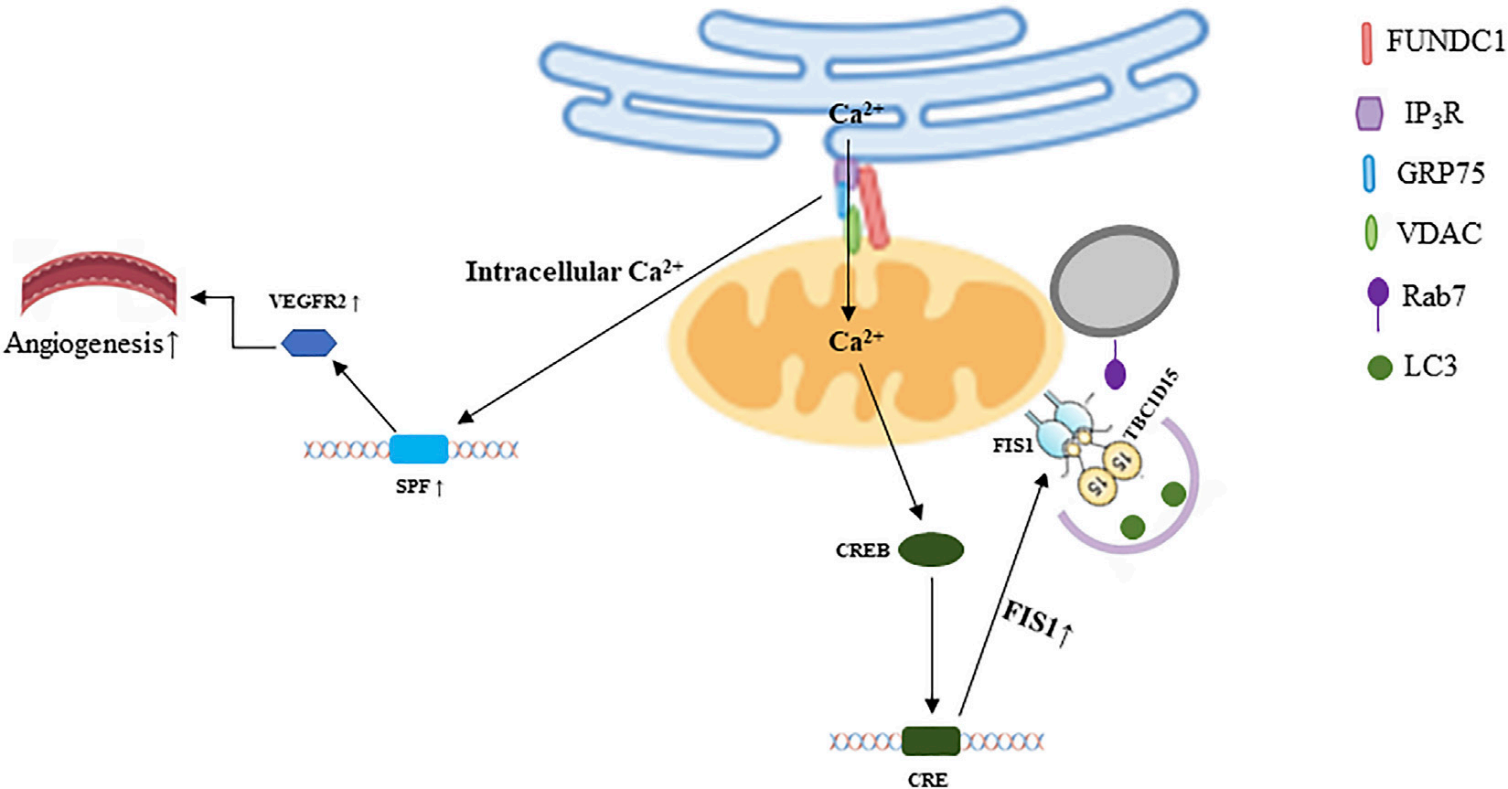
FUNDC1 mediated crosstalk among mitochondria and other organelles. The illustration was created by Figdraw (www.Figdraw.com).

The most typical membrane contact site is the interaction between mitochondria and ER, known as mitochondria-associated ER membranes (MAMs) or mitochondria-ER contacts (MERCs), it maintains the homeostasis of Ca^2+^ level and lipid metabolism in two organelles (Rowland and Voeltz, 2012; Giacomello and Pellegrini, 2016). FUNDC1-mediated MAMs are involved in the transportation of calcium. Calcium is transported to mitochondria from ER through the inositol 1,4,5-triphosphate receptor (IP3R)-glucose regulated protein 75 (Grp75)-voltage-dependent anion channel (VDAC) complex, FUNDC1 which interacts with IP3R2 is responsible for facilitating MAMs stabilization. The cAMP-responsive element binding protein (CREB) is phosphorylated at S133 site due to mitochondrial retrograde calcium signal, which inducing CREB nuclear translocation, and then the transcription of fission one protein (FIS1) is activated. Subsequently, enhanced FIS1 promotes both mitochondrial fission and mitophagy (Wu et al., 2017b; Muñoz and Zorzano, 2017), as shown in Figure 2. Moreover, it has been suggested that FUNDC1 may maintain mitochondrial calcium homeostasis *via* the interaction between FUNDC1 and receptor subunit of human SCF (SKP1/cullin/F-box protein) ubiquitin ligase complex (FBXL2) (Ren et al., 2020).

FUNDC1-dependent MAMs are involved in the development of multiple diseases. For example, diabetes mellitus inhibits the activity of AMP-activated protein kinase (AMPK), which promotes the formation of FUNDC1-mediated MAMs, and then inducing mitochondrial dysfunction, leading to cardiomyopathy (Wu et al., 2019a). AMPK-FUNDC1-MAMs axis is also involved in the development of diabetic nephropathy (DN) (Wei et al., 2020). Excessive Ca^2+^ releasing from ER into mitochondria leads to cardiac damage and heart failure when FUNDC1 interacts with IP3R2 (63). Moreover, if the formation of FUNDC1-mediated MAMs is disrupted under angiogenic condition, the level of serum response factor (SRF) and vascular endothelial growth factor 2 (VEGFR2) will be downregulated due to the dyshomeostasis of Ca^2+^, which reducing the VEGF-induced angiogenesis (Wang et al., 2021b), as shown in Figure 2. Some studies documented that FUNDC1-mediated mitophagy could improve cerebral ischemia *via* inhibiting NLRP3 inflammasome. And the level of NLRP3 would be increased when FUNDC1 was knocked out, then inflammatory response was exacerbated, lung cells were further injured (Pan et al., 2021; Zheng et al., 2021). Furthermore, the activation of NLRP3 inflammasome compromises the recovery of HFD-induced vascular impairment (Elshaer et al., 2017; Mohamed et al., 2020). Considering that FUNDC1 can regulate NLRP3, and FUNDC1-mediated MAMs can promote angiogenesis, whether there is a link between FUNDC1-mediated MAMs and NLRP3 is worthy of mention. MAMs may be potential drug targets. For example, capsaicin improves DN *via* activating transient receptor potential cation channel subfamily V member 1 (TRPV1). The molecular mechanism is that AMPK is activated by transient Ca^2+^ level due to the activation of TRPV1, causing the reduction of FUNDC1, which downregulates the formation of MAMs (Wei et al., 2020).

#### 4.4.2 Lysosome

Mitochondria and lysosome are important organelles to maintain cell homeostasis. Their dysfunction or interaction disorders are related to neurodegeneration and other human diseases (Audano et al., 2018; English and Hughes, 2019). The role of mitochondria-lysosome contacts in the function of mitochondria and lysosome has received increased attention in recent years. Apart from regulating mitochondrial fission, Wong et al. (2018) discovered that the morphology of lysosome would be impaired if the mitochondria-lysosome contact could not untether.

Rab7 is a small G protein belonging to the Rab family and is present on the lysosome, ER, and mitochondrial membranes (Jimenez-Orgaz et al., 2018). It is mainly involved in membrane trafficking (Progida et al., 2010). Furthermore, Rab7 is involved in regulating mitochondria-lysosome contact (Wong et al., 2018). Activated Rab7 maintains mitochondrial-lysosome contact, while inactivated Rab7 makes mitochondrial-lysosome contact dissociated. The activation of Rab7 is regulated by TBC1D15, which belongs to the TBC (Tre-2/Bub2/Cdc16) domain family and function as a GTPase-activating protein (GAP) for Rab GTPases (Chen et al., 2017c; Wong et al., 2018). TBC1D15 could be recruited to mitochondria by FIS1 (Onoue et al., 2013). And then, TBC1D15 makes Rab7-GTP translate to Rab7-GDP, which cannot bind to the lysosome, ultimately promoting the dissociation between mitochondria and lysosome (Wong et al., 2018). Autophagy is inhibited in a variety of diseases, but the formation of autophagosomes is normal or even enhanced. If there are not enough or normal functional lysosome to receive autophagosomes, autophagosomes will not be degraded and may lead to cytotoxicity (Cuervo, 2011). In view of that, Yu Wenjun and his colleagues proved that TBC1D15/FIS1/Rab7 pathway exerted protective effects on the function and morphology of infarct heart *via* untethering mitochondria-lysosome contact (Yu et al., 2020).

TBC1D15 could modulate autophagosome biogenesis *via* interacting with LC3/GABARAP family members (Yamano et al., 2014). And FIS1 regulates mitochondrial fission by recruiting lysosome toward mitochondria and is primarily involved in the clearance of damaged mitochondria (Kleele et al., 2021). Moreover, FUNDC1 can sense the inner mitochondrial stress *via* interacting with OPA1(38) and increase the level of Ca^2+^ in mitochondria, which is a sensor that promotes transcription of FIS1(64). Whether these proteins can coordinate fission and mitophagy and whether these processes could happen continuously, the mechanisms in integrating these processes are unclear. Thus, further in-depth studies are needed to explore whether the FUNDC1/FIS1/TBC1D15/Rab7 pathway participates in regulating mitochondria-lysosome contact, as shown in Figure 2.

## 5 FUNDC1 and diseases

### 5.1 Ischemia-reperfusion (I/R) injury

**FIGURE 3:**
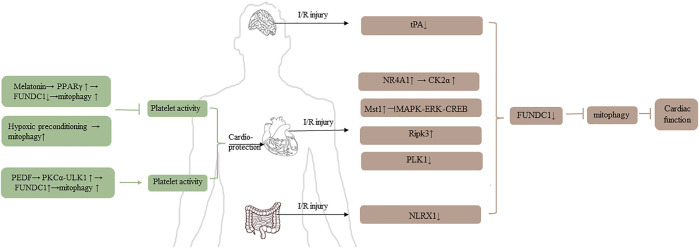
The role of FUNDC1 in I/R injury.

Ischemic disease, which has high disability and mortality, especially heart and brain ischemia, occurs when blood vessels are blocked (Zhang et al., 2021). Although reperfusion is necessary to recover cell function, it can also provoke activation of deleterious processes to impair cell function (Kalogeris et al., 2016). Numerous studies demonstrated that mitophagy played a vital role in the physiopathology and treatment of I/R injury. The details are shown in Figure 3.

Platelet reactivity is related to mitochondrial mass and function, and it is crucial for I/R injury (Zhang et al., 2017; Davizon-Castillo et al., 2019). There are two studies to clarify the relationship between mitochondria and platelet. Zhou et al. (2017a) pointed out that the level of peroxisome proliferator-activated receptor γ (PPARγ) was downregulated in platelets under I/R injury condition, and then FUDNC1-mediated mitophagy was activated. Subsequently, the platelet was activated, which promoting the block of microcirculation, finally causing cardiomyocyte death, excessive inflammation response, and heart dysfunction. According to the report, melatonin can reverse heart injury *via* promoting the level of PPARγ, PPARγ-FUNDC1-mitophagy pathway could be an essential strategy for treating I/R injury. Coincidentally, Zhang et al. (2016), Zhang et al. (2017) used conditional knockout mice specifically lacking FUNDC1 in their platelets and found that hypoxic preconditioning had a cardioprotective effect because hypoxic induced mitophagy causing extensive mitochondrial degradation in platelets, and then compromising the activation of platelet, reducing the I/R injury. It seems that mitophagy could upregulate or downregulate the activity of platelet under different conditions. It is unclear whether the contradictory results are due to inconsistent intervention methods in the two experiments or whether they suggest other pathways other than FUNDC1- mediated mitophagy affecting platelet activity.

From the perspective of cardiac ischemia studies, it is generally believed that the regulation of mitophagy may be a potential therapy for cardioprotective effect. Zhou Hao and his colleagues’ study revolved around nuclear receptor subfamily four group A member 1 (NR4A1) and FUNDC1. Increased NR4A1 was observed in cardiac I/R model, upregulated NR4A1 induced FUNDC1 phosphorylation *via* activating CK2α, increased CK2α was harmful for mitochondria, and then mitophagy was suppressed, ultimately impairing cardiac function. Moreover, Zhou et al., (2018a), Zhou et al. (2018b) explored the relationship between NR4A1 and FUNDC1 in alcohol-related liver disease (ARLD). They revealed that NR4A1 was increased after alcohol treatment, upregulated NR4A1 could promote the disassociation of DNA-dependent protein kinase catalytic subunit (DNA-PKcs) and Ku80. Subsequently, disassociated DNA-PKcs bound to p53, activated p53 promoted the upregulation of CK2. Same results as the cardiac I/R model, FUNDC1-mediated mitophagy was suppressed, ultimately leading to the progression of ARLD (Zhou et al., 2019).

Mammalian STE20-like kinase 1 (Mst1) which is a negative regulator of cardiac function is upregulated in acute I/R injury model, inhibits MAPK-ERK-CREB pathway, consequently suppresses the level of FUNDC1, thus FUNDC1-mediated mitophagy is defective (Yu et al., 2019). Receptor-interacting protein kinase 3 (Ripk3) is another factor to damage cardiac function. Upregulated Ripk3 can interact with FUNDC1, inhibiting mitophagy, leading to caspase9-mediated apoptosis (Zhou et al., 2017b). The level of polo-like kinase 1 (PLK1) is downregulated in cardiac I/R injury model, but overexpression of PLK1 can relieve myocardial damage *via* p-AMPK/FUNDC1-mediated mitophagy (Mao et al., 2021). Pigment epithelial-derived factor is a protective factor for acute myocardial infarction. Under hypoxia condition, it can interact with PEDF-R, and then active PKC-α through increasing the level of palmitic acid and diacylglycerol. The activated PKC-α can promote FUNDC1-mediated mitophagy, which depends on ULK1 pathway, thus protecting cardiomyocytes (Li et al., 2018). In addition to myocardial ischemia, ULK1-FUNDC1-mediated mitophagy is also the mechanism of renoprotection using ischemia preconditioning in ischemic acute kidney injury (Wang et al., 2020).

mTORC1-ULK1-FUNDC1-mitophagy is an important axis to reduce ischemic injury. Electroacupuncture can not only attenuate myocardial ischemia-reperfusion injury but also cerebral ischemia-reperfusion injury *via* mitophagy inhibited by mTORC1-ULK1-FUNDC1 pathway (Mao et al., 2020; Xiao et al., 2020; Tian et al., 2021). By contrast, Cai Ying and his colleagues investigated that tissue-type plasminogen activator (tPA) was decreased in cerebral I/R model, causing FUNDC1 degraded and mitophagy inhibited, while exogenous tPA, which could induce FUNDC1-mediated mitophagy *via* phosphorylating AMPK, could reduce I/R injury (Cai et al., 2021).

Furthermore, FUNDC1-mediated mitophagy is also an underlying physiopathology mechanism in intestinal I/R injury. Li Shaoqin revealed that NOD-like receptor X1 (NLRX1) was decreased in intestinal I/R model, and the phosphorylation of FUNDC1 was enhanced. Thus, FUNDC1 could not interact with non-neuronal SNAP25-like protein homolog one and 2 (NIPSNAP one and 2), resulting in the suppression of mitophagy, ultimately contributing to intestinal morphological damage (Li et al., 2021).

### 5.2 Cancers

High level of FUNDC1 promotes oxidative bioenergetics and supports tumor cell proliferation and growth (Cappellini et al., 2020). It might be an independent prognostic factor for overall survival and disease-free survival in patients with cervical cancer (Hou et al., 2017). Hydrogen peroxide is a regulator to increase the level of FUNDC1 in laryngeal cancer cells, mainly through activating ERK1/2 (Hui et al., 2019). Furthermore, FUNDC1-involved MAMs can promote calcium transport to cytoplasm, making nuclear factor of activated T cells 1 (NFATC1) dephosphorylated, and then Bmi1 polycomb ring finger oncogene (BMI1) is upregulated, thus promoting the progression of breast cancer (Wu et al., 2019b). FUNDC1 promotes the growth of hepatocellular carcinoma (HCC) tissues in the late stage of the disease (Li et al., 2019c), and the high level of FUNDC1 might also be a risk factor for HCC patients. However, Li Wenhui found that FUNDC1-mediated mitophagy partially inhibited the release of mtRNA and mtROS produced by damaged mitochondria, thereby preventing inflammasome hyperactivated, suppressing the initiation of HCC in early-stage (Li et al., 2019c). These inconsistent results might be interpreted using the dynamic function of autophagy. Micro-137 is a protective factor for breast cancer because it can target FUNDC1 to inhibit the occurrence of mitophagy (Hu et al., 2020).

### 5.3 Metabolic-related diseases

Mitochondria are energy factories, participating in the occurrence of multiple metabolic-related events. In recent years, mitophagy, which is regarded as the key mechanism for mitochondrial quality control and function, has received increased attention in metabolic-related diseases.

Like the global knockout mice, Wu Hao’s study showed that severe mitochondrial abnormality, aggregated inflammation, obesity phenotypes, and insulin resistance phenotypes were observed when animals were fed high fat diet in the adipose tissue specific FUNDC1 knockout mice model. The mechanism is mainly related to the accumulated ROS in damaged mitochondria, because ROS can activate MAPK, which leads to white adipose tissue insulin resistance and systematic insulin resistance induced by elevated tumor necrosis factor (TNF), interleukin 6 (IL6) and downregulated adiponectin gene (ADIPOQ) (Wu et al., 2019c). However, in skeletal-muscle specific FUNDC1 knockout mice, the obesity and systemic insulin resistance induced by high-fat diet are improved. Fu et al. (2018) argued that FUNDC1 deficiency in skeletal muscle could evoke a retrograde response in muscle that promoted the thermogenic remodeling of adipose tissue through increasing the level of fibroblast growth factor 21(FGF21). The two seemingly contradictory results above suggest that FUNDC1 in mitochondria of diverse tissues may have different physiological roles, and FUNDC1 may also play other roles besides mediating mitophagy. It seems to be worth further investigating the phenotypes of mice in which FUNDC1 is knocked out simultaneously in skeletal muscle and adipose tissue.

## 6 Conclusion

**FIGURE 4:**
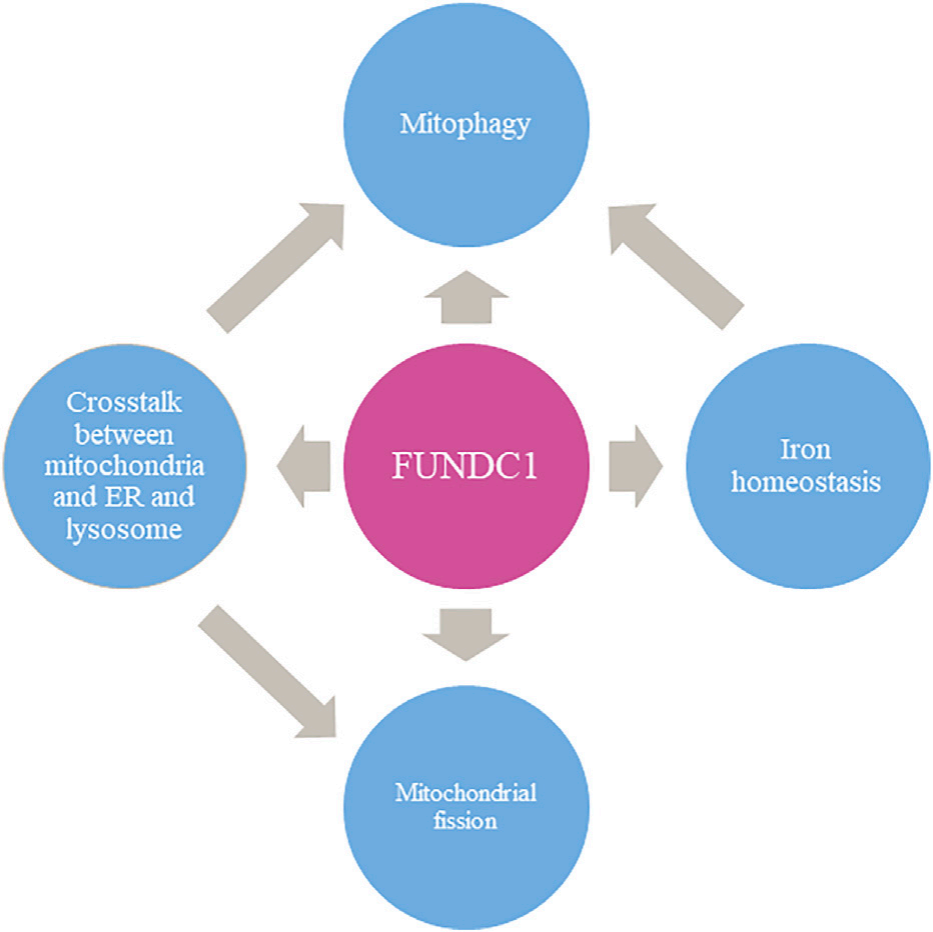
The multi-faced role of FUNDC1 in mitochondrial events.

FUNDC1 is a crucial molecule which contains multiple sites performing different functions. FUNDC1 is mainly involved in mitophagy. On this basis, it is also involved in promoting mitochondrial division, maintaining iron homeostasis in cells, removing unfolded proteins, and maintaining cell functions through interacting with endoplasmic reticulum or lysosome.(as shown in Figure 4). Further investigation of the mechanism of the crosstalk are still needed to help combat human diseases. FUNDC1 is involved in the physiological and pathological processes of I/R injury, cancers, and metabolic-related diseases. It seems clear that targeting FUNDC1 is a potential therapeutic option for numerous diseases related to mitochondria abnormality, although there are currently few small molecule drugs targeting FUNDC1.
